# Operative Versorgungsstrategien bei periprothetischen Femurfrakturen Typ Vancouver B

**DOI:** 10.1007/s00132-025-04613-y

**Published:** 2025-02-18

**Authors:** Christian Ries, Patrick Gerhardt, Peter Helwig, Holger Bäthis, Stephan Kirschner, Tim Rolvien, Frank Timo Beil

**Affiliations:** 1 Klinik für Unfallchirurgie und Orthopädie, Lehrstuhl für Orthopädie, Universitätsklinikum Hamburg-Eppendorf, Hamburg, Deutschland; 2 Klinik für Orthopädie und Unfallchirurgie, Agaplesion Markus Krankenhaus, Frankfurt am Main, Deutschland; 3 Klinik für Orthopädie und Unfallchirurgie, Klinikum Heidenheim, Heidenheim, Deutschland; 4 Klinik für Orthopädie, Unfallchirurgie und Sporttraumatologie, Klinikum Köln-Merheim, Lehrstuhl der Universität Witten-Herdecke, Köln, Deutschland; 5 Klinik für Orthopädie, ViDia-Kliniken, Karlsruhe, Deutschland; 6 AE-Komitee „Frakturendoprothetik und periprothetische Frakturen“, Deutsche Gesellschaft für Endoprothetik (AE), Freiburg, Deutschland

**Keywords:** Hüfttotalendoprothese, Periprothetische Femurfraktur, Prothesenrevision, Osteosynthese, Osteoporose, THA, Periprosthetic fracture, Revision THA, Osteosynthesis, Osteoporosis

## Abstract

**Hintergrund:**

Durch den demographischen Wandel ist eine weitere Zunahme von endoprothetischen Versorgungen anzunehmen. Entsprechend ist, wie bereits in den letzten Jahren zu beobachten, mit einem weiteren Anstieg periprothetischer Frakturen zu rechnen. Die periprothetische Fraktur ist mittlerweile der dritthäufigste Grund für eine Revisionsoperation nach Implantation einer Hüfttotalendoprothese.

**Ziel der Arbeit:**

Unter Berücksichtigung der bekannten Risikofaktoren für periprothetische Femurfrakturen (PPF) werden die Versorgungsstrategien der periprothetischen Frakturbehandlung auf Grundlage der aktuellen Erkenntnisse evaluiert, um Empfehlungen für die Praxis aussprechen zu können.

**Material und Methoden:**

Narratives Review.

**Ergebnisse:**

Die Literatur ist sehr heterogen und für viele Aspekte fehlt die Evidenz. Basis vieler Behandlungsempfehlungen bilden nicht randomisierte Studien mit geringer Patientenanzahl. Die Mortalität nach PPF ist unabhängig von der gewählten Therapie hoch. Das Alter und die Knochenqualität spielen eine Rolle bei der Versorgungstrategie und bei den heterogenen Ergebnissen. Für die Schaftverankerung bei Wechseloperationen nach proximaler PPF werden zementierte und zementfreie Schäfte in der Literatur gleichermaßen verwendet. Signifikante Ergebnisunterschiede zeigen sich nicht. Ein propagierter Vorteil von zementfreien Schäften in modularer Ausfertigung für diese Versorgung wird von der Literatur aktuell nicht gestützt.

**Schlussfolgerungen:**

Unter Berücksichtigung der Umfeldfaktoren und Komorbiditäten empfiehlt sich ein individueller Ansatz bei der Versorgung PPF. Beim geriatrischen Patienten sollte zur Vermeidung von Komplikationen postoperativ eine Vollbelastung der unteren Extremität angestrebt werden.

## Abstract (en)

**Background:**

The demographic shift is expected to lead to a further increase in the number of hip joint replacements. Accordingly, as has already been observed in recent years, a further increase in periprosthetic femoral fractures (PPF) is to be expected. PPF is now the third most common reason for revision surgery after hip arthroplasty.

**Objectives:**

Taking into account the known risk factors for PPF, fracture treatment strategies are evaluated based on current evidence in order to make recommendations for practice.

**Methods:**

Narrative review.

**Results:**

Overall, the literature is very heterogeneous and evidence is lacking for many aspects. Numerous recommendations are based on non-randomized studies with low patient count. Mortality after PPF is high regardless of the treatment chosen. Age and bone quality influence the partly heterogeneous results and play a role in the treatment strategy. The use of both cemented and uncemented stems in revision surgery due to proximal PPF is frequently described in the literature. There are no significant differences in terms of outcome. The advantage of uncemented modular stems is currently not supported by the literature.

**Conclusions:**

An individualized approach to the treatment of PPF is recommended, considering environmental factors and comorbidities. In geriatric patients, full weight-bearing of the lower extremity should be aimed for postoperatively to avoid complications.

Die periprothetische Femurfraktur ist eine schwerwiegende Komplikation nach endoprothetischem Hüftgelenkersatz. Verschiedene Faktoren beeinflussen das Frakturmanagement. Die Therapiekosten dieser Verletzungen sind hoch. Geringe Komplikationsraten sind zum Schutz der Patienten erstrebenswert. Diese Übersichtsarbeit beschreibt Versorgungsstrategien der periprothetischen Frakturbehandlung unter besonderer Berücksichtigung des oftmals betagten Patientenkollektivs.

Der endoprothetische Gelenkersatz ist die Therapie der Wahl bei fortgeschrittener Degeneration des Hüftgelenkes und wurde aufgrund des therapeutischen Erfolges nicht zu Unrecht von Learmonth und Kollegen in ihrem Literaturreview als die „Operation des Jahrhunderts“ bezeichnet [1]. Das Endoprothesenregister Deutschland (EPRD) verzeichnete für das Jahr 2022 mehr als 175.000 primäre Implantationen von Hüftendoprothesen. Der überwiegende Anteil dieser Implantate wurde – konstant zu den vorherigen Jahren – zementfrei eingebracht (77,2 %, Ø 67 Jahre bei Primärimplantation) [2]. Der Anteil an Hybridfixierungen wurde für 2022 mit 17,9 % beziffert, wobei die Patienten in diesem Kollektiv im Durchschnitt bereits 79 Jahre alt waren [2]. Anhand der Registerdaten zeigt sich insbesondere im frühen Nachuntersuchungszeitraum (< 6 Monate) eine erhöhte Ausfallwahrscheinlichkeit für zementfreie Schaftfixierungen bei Patienten über 75 Jahre [2].

Studien konnten zeigen, dass eine reduzierte Knochenqualität und altersassoziierte Veränderungen innerhalb des proximalen Femurs die initiale Implantatstabilität beeinträchtigen. Eine negative Auswirkung auf die knöcherne Osseointegration des Prothesenschaftes ist anzunehmen, wodurch das Risiko der Schaftmigration und letztendlich auch die Ausfallwahrscheinlichkeit konsekutiv steigen [3, 4]. In einem Kollektiv von 269 Patienten (> 70 Jahre), welche zur Implantation einer Hüftendoprothese geplant wurden, zeigte sich bei 41 % eine Osteopenie. Innerhalb des Kollektivs litten zusätzlich 18 % an einer Osteoporose, wovon 73 % zuvor nicht diagnostiziert waren [5]. Bereits osteopene Knochenverhältnisse (T-Score < − 1) erhöhen das intraoperative Frakturrisiko um das 5fache [6]. In der Betrachtung des Risikos einer periprothetischen Femurfraktur (PPF) bei der Primärendoprothetik ist eine intraoperative Fraktur bei zementfreien Schäften 14-mal häufiger zu beobachten [7].

Weiterhin konnten Abdel et al. auch im postoperativen Verlauf über die Jahre hinweg ein kontinuierlich ansteigendes Frakturrisiko nach zementfreier Primärimplantation aufzeigen [7]. Das weibliche Geschlecht und das Alter sind weitere Risikofaktoren für eine PPF [5, 7]. Die PPF sind mittlerweile nach der Lockerung (22,7 %) und der Infektion (16,4 %) der dritthäufigste Grund (15,9 %) für eine Revisionsoperation [2]. Die Einteilung der PPF erfolgt anhand gebräuchlicher Klassifikationen, welche neben der Frakturlokalisation auch die verbliebene Fixierung der femoralen Komponente berücksichtigen. Die Vancouver-Klassifikation unterteilt die Frakturen in die Typen A bis C.

Während Typ-A-Frakturen die Lokalisation im Bereich der Trochanteren beschreibt, befindet sich die Fraktur bei Typ B auf Höhe des Prothesenschaftes. Hier ist insbesondere zwischen stabiler/fester (Typ B1) und instabiler/gelockerter Komponente (Typ B2/3) zu unterscheiden. Die Typ-C-Frakturen sind unterhalb des fest inserierten Schaftes lokalisiert. Die Frakturtypen A bis C sind analog zum neueren Unified Classification System (UCS), welches darauf abzielt, eine einheitliche Klassifikation für alle Arten von periprothetischen Frakturen zu ermöglichen. PPF vom Typ Vancouver B2 und B3 werden eher bei älteren Patienten (> 75 Jahren) beobachtet. Die höchste Inzidenz wird für Patienten älter als 80 Jahre beschrieben. Hier zeigt sich für die Subtypen B1, B2 und C ein Altersgipfel zwischen 80 und 89 Jahren [8]. Eine ähnliche Zunahme der Subtypen A und B3 zeigt sich zwischen 70 und 79 Jahren, wenngleich dieser weniger deutlich ist [8]. Chatziagorou et al. konnten in ihrer Arbeit Typ-B2-Frakturen vermehrt bei primär nicht zementierter Schaftfixierung aufzeigen. Der Subtyp C ist hingegen häufiger mit einer primär zementierten Schaftfixierung assoziiert [8]. In einer aktuellen Arbeit von Ritter et al. zeigte sich im analysierten Kollektiv bei 45 % der PPF eine Osteoporose [9].

Durch den demographischen Wandel ist eine weitere Zunahme von endoprothetischen Versorgungen anzunehmen. Entsprechend ist, wie über die letzten Jahre ersichtlich, ein Anstieg an PPF zu erwarten [2]. Als Risikofaktoren für eine PPF werden neben Alter, Geschlecht und Osteoporose auch inflammatorische Arthritiden und lokale, implantatassoziierte Osteolysen und Knochendefekte genannt [10]. Das kombinierte Risiko für eine Reoperation oder für das Versterben innerhalb des ersten Jahres nach einer PPF wird mit bis zu 24 % angegeben [11]. Die Therapiekosten dieser Verletzungen sind mitunter enorm [12, 13]. Das Ziel sollte daher eine möglichst geringe Komplikationsrate bei der Revisionsoperation sein, um letztendlich nicht nur vorranging den Patienten zu schützen, sondern auch sozioökonomisch kosteneffektiv zu arbeiten. Unter Berücksichtigung all dieser Aspekte erscheint es sinnvoll, gerade für die betagten Patienten die Versorgungsstrategien der periprothetischen Frakturbehandlung zu evaluieren und Empfehlungen für die Praxis abzuleiten.

## Versorgungsstrategien bei PPF Typ Vancouver B

Für die Klassifikation der PPF sind, wie eingangs erwähnt, verschiedene Systeme etabliert. Da sich gegenwärtig die meisten Publikationen auf die Vancouver-Klassifikation [14] beziehen, wird auch in der vorliegenden Arbeit diese Klassifikation als Referenz herangezogen. Mitunter kann es schwierig sein, präoperativ anhand der Bildgebung zu beurteilen, ob die femorale Komponente gelockert ist oder nicht. Im Rahmen der Anamnese sollten bereits vorbestehende Beschwerden, welche auf eine Schaftlockerung hinweisen könnten, erfragt werden. Im Zweifelsfall sollte sich der Behandelnde darauf vorbereiten in situ eine gelockerte Prothese vorzufinden (Typ Vancouver B2). Die Einteilung der PPF zum Subtyp B3 – gelockerte Prothese bei schlechter Knochenqualität – erfolgt oftmals subjektiv und lässt sich nicht zwingend anhand der präoperativen Bildgebung ableiten. Bei PPF vom Typ Vancouver B ist die operative Revision als Goldstandard anzusehen. Verschiedene Faktoren beeinflussen das Frakturmanagement. Hierbei spielen die Frakturlokalisation in Relation zum Implantat, die Art der primären Schaftfixierung als auch die Knochenqualität eine übergeordnete Rolle. Im Allgemeinen gibt es zwei Versorgungsstrategien bei PPF. Zum einen die alleinige Osteosynthese mit Erhalt der femoralen Schaftkomponente. Zum anderen den Schaftwechsel mit oder ohne zusätzliche Osteosynthese.

## Operatives Vorgehen – Schaftrevision mit oder ohne Osteosynthese oder doch nur die Osteosynthese?

**Abb. 1:**
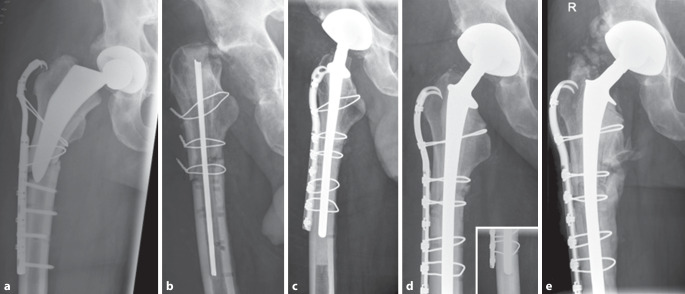
73 Jahre, männlich, Body-Mass-Index 29,9 kg/m^2^. **a** Osteosyntheseversagen wenige Wochen nach im Ausland versorgter subtrochantärer periprothetischer Querfraktur Typ Vancouver C. **b** Nachweis von Staphylococcus epidermidis im Gelenkpunktat. Explantation, Debridement und Anlage einer Girdlestone-Situation. Die Stabilisation des osteotomierten proximalen Femurs erfolgte mittels 1,5 mm Drahtcerclagen sowie durch eine intramedulläre Schienung des Femurschaftes (durch K‑Drähte verstärkter, antibiotikahaltiger Zementspacer). Intraoperativ Bestätigung des Nachweises von Staphylococcus epidermidis. **c**/**d** Röntgenverlaufskontrolle des Hüftgelenkes axial und a.p. nach zweizeitiger Reimplantation im 6‑wöchigen Intervall einer zementfreien tripolaren Gelenkpfanne, eines zementierten Revisionsschaftes sowie der Osteosynthese mittels Krallenplatte und Cable Cerclagen zur additiven Stabilisation der subtrochantären Querfraktur. Perioperativ erfolgte für 12 Wochen die resistogrammgerechte Antibiose. **e** Röntgenverlaufskontrolle 2 Jahre postoperativ mit Nachweis der Frakturkonsolidierung ohne Hinweis auf eine Materiallockerung und/oder ein Materialversagen. Der Patient ist beschwerdefrei und ohne Hilfsmittel eigenständig mobil. Nebenbefundlich heterotope Ossifikationen (Brooker-Stadium II)

**Abb. 2:**
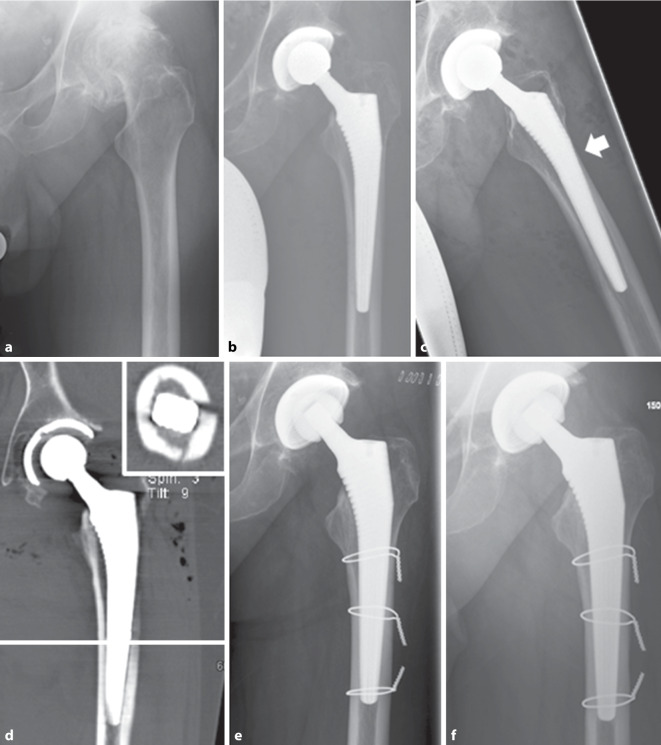
**a** 58 Jahre, männlich. Fortgeschrittene destruierende Koxarthrose. **b**/**c** Postoperative Röntgenkontrolle a. p. und axial nach Lauenstein mit dem V. a. eine Sinterung bei periprothetischer Femurfraktur (PPF, *Pfeil*) unmittelbar postoperativ. **d** Postoperatives CT (koronar und axial; die *weiße Linie* entspricht der Höhe in der axialen Projektion) mit Bestätigung des gesinterten Schaftes (PPF Vancouver-Typ B2). **e** Postoperative Kontrolle nach Revisionsoperation mit offener Reposition und Osteosynthese (1,5 mm Drahtcerclagen) sowie zementfreiem Schaftwechsel. Sicherungscerclage auf Höhe der Schaftspitze mit ausreichend Abstand zur anatomisch reponierten und fixierten Fraktur. Postoperativ 6 Wochen Entlastung der linken unteren Extremität. **f** In der Nachuntersuchung 1 Jahr nach der Revision zeigt sich eine konsolidierte Fraktur bei unveränderter Schaftlage

Bei PPF mit nichtgelockerter femoraler Komponente (Vancouver Typ B1) wird die offene Reposition und interne Fixierung (ORIF) gegebenenfalls mit Cerclagen und additiver winkelstabiler Plattenosteosynthese angestrebt [15]. Zeigt sich ein gelockerter femoraler Schaft (Vancouver Typ B2/B3), wird oftmals die osteosynthetische Versorgung der Fraktur z. B. mittels Cerclagen (mindestens 2 Stück) und die Schaftrevision unter Verwendung eines diaphysär verankernden zementfreien Revisionsschaftes empfohlen [16, 17]. Hierbei ist die ausreichende Überbrückung der Frakturzone sicherzustellen (mindestens zwei Femurschaftbreiten bzw. 4–6 cm). Der femorale Isthmus muss hierfür erhalten sein, um letztendlich bei zementfreier Revision eine ausreichende „Press-fit“-Stabilität im distalen Fragment zu erzielen. Liegt eine Querfraktur des Femurschaftes vor, sollte additiv eine überbrückende Abstützung z. B. mittels Plattenosteosynthese erfolgen, um die Biegekräfte auf den Prothesenschaft zu reduzieren und die Frakturkonsolidierung zu begünstigen ([16]; Abb. 1a–e). Die postoperative Belastung der versorgten unteren Extremität ist entsprechend zu adaptieren und erfolgt häufig mit einer Teilbelastung von 20 kg für 6–12 Wochen ([16, 18]; Abb. 2a–f).

**Abb. 3:**
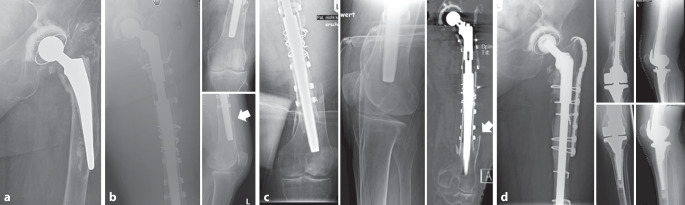
84 Jahre, weiblich. **a** Aseptisch gelockerte zementierte Prothese (Primärimplantation vor 17 Jahren) mit distaler Schaftspitzenmigration und Destruktion der lateralen Femurkortikalis und konsekutiver Fraktur (Vancouver-Typ B3). Rarefizierte Knochenstruktur. **b** Postoperative Kontrolle nach zementfreiem, modularem Schaftwechsel mit additiven Cerclagen. Bereits hier ist distal die periprothetische Anschlussfraktur (*Pfeil*) ersichtlich. **c** Postoperative Röntgenkontrolle 16 Tage nach der initialen Revisionsoperation. Zunehmende Dislokation (*Pfeil*) der Fraktur (Röntgen und CT). **d** Postoperative Kontrolle nach erneuter Revision mit Wechsel auf eine zementierte Durchsteckprothese mit Implantation einer gekoppelten Knieendoprothese sowie additiver Krallenplattenosteosynthese. Postoperativ schmerzadaptierte Vollbelastung der Extremität

Bei der Verwendung von zementfreien Schäften besteht letztendlich nicht nur nach primärer Endoprothetik, sondern auch im Revisionsfall das Risiko einer Nachsinterung des Schaftes [19, 20]. Hierbei wird eine Sinterung über 5 mm als kritisch betrachtet [21]. Wenngleich zementfreie Revisionsschäfte mit einem erhöhten Risiko der Nachsinterung assoziiert sind, so wird die 10-Jahres-Standzeit mit bis zu 98 % angegeben [20, 22–24]. Allerdings sollten patientenspezifische Risikofaktoren mit in die therapeutische Planung einbezogen werden, um Komplikationen zu vermeiden (Abb. 3a–d). Das Risiko für eine intraoperative Fraktur ist insbesondere bei Femurschaftkonfigurationen mit breitem Markraum und ausgedünnter Kortikalis (Typ Dorr C) signifikant erhöht [25]. Ist keine „Press-fit“-Stabilität zu erreichen, sollte daher die zementierte Schaftfixierung erwogen werden. Während das Frakturrisiko bei der zementfreien Primärimplantation bereits erhöht ist, beträgt das Frakturrisiko bei zementfreien Revisionseingriffen bis zu 21 % (zementiert 3,6 %) [26].

Khan et al. [27] konnten in ihrem systematischen Literaturreview 510 PPF vom Typ B2 und B3 einschließen. Von den eingeschlossenen B2-Frakturen (*n* = 343) erhielten 86,8 % eine Schaftrevision (82 zementiert vs. 153 zementfrei; 63 „nicht spezifiziert“) mit oder ohne Osteosynthese. 12,6 % wurden alleinig osteosynthetisch versorgt. Die Revisionsraten betrugen für die Schaftrevision 12,4 % bzw. 13,3 % für die Osteosynthese. Die Re-Revisionsrate in Anlehnung an die Schaftfixierung (zementiert vs. zementfrei) wurde mit 13,4 % bzw. 12,4 % beziffert. Die B3 Frakturen (*n* = 167) wurden ebenfalls vorwiegend mittels Revision des Prothesenschaftes versorgt (95,8 %; 49 zementiert vs. 90 zementfrei; 21 „nicht spezifiziert“). Nur in 4,8 % der Fälle erfolgte ausschließlich eine Osteosynthese. Die Revisionsrate bei alleiniger Osteosynthese nach B3-Fraktur war fast doppelt so hoch (28,6 % vs. 14,4 %). Die Re-Revisionsrate lag in diesem Kollektiv bei 16,3 % für die zementierten und bei 13,3 % für die zementfreien Schäfte. Am häufigsten wurden zementfreie Schäfte mit distaler diaphysärer Verankerung verwendet (nicht modular Typ Wagner SL Schaft, Fa. Zimmer Biomet, Warsaw, Indiana, USA). Die Rate an Schaftsinterungen betrug 5,2 %. Distal verriegelte Schäfte kamen in 8,5 % der Fälle zur Anwendung.

Die häufigsten Gründe für eine Re-Revision waren die Re-Fraktur, die Infektion sowie die Schaftsinterung. Die alleinige Osteosynthese ohne Schaftrevision bei B2- und B3-Frakturen zeigte in der vorliegenden Studie schlechtere Ergebnisse. Dies deckt sich mit anderen Arbeiten, in denen für die alleinige Osteosynthese bei B2/B3-Frakturen ebenfalls signifikant höhere Revisionsraten beschrieben werden, als bei Schaftrevisionen mit oder ohne Osteosynthese (22,0 % vs. 13,5 %) [15]. Chatziagorou et al. [15] beschreiben zudem, dass postoperativ nach alleiniger Osteosynthese in 64 % der Fälle keine volle Belastung der Extremität freigegeben wurde. Die Autoren schlussfolgerten, dass sich hierin womöglich die reduzierte Frakturstabilisation im Vergleich zur Schaftrevision (87,5 % Vollbelastung postoperativ) widerspiegelt [15]. Insbesondere bei betagten Patienten sollte allerdings auch zur Vermeidung internistischer Komplikationen die frühe postoperative Mobilisation (Vollbelastung nach abgeschlossener Wundheilung) erfolgen. Eine Entlastung der unteren Extremitäten ist von älteren Patienten oftmals koordinativ oder auch kognitiv nicht einzuhalten.

Nichtsdestotrotz kann auch die alleinige Osteosynthese in ausgewählten Fällen eine therapeutische Option sein. Di Martino et al. [28] analysierten in ihrer Metaanalyse 1629 Patienten (564 ORIF vs. 1065 Schaftrevisionen). Die alleinige Osteosynthese zeigte neben einem geringeren intraoperativen Blutverlust, einer kürzeren Operationsdauer auch einen kürzeren Krankenhausaufenthalt. Patienten mit signifikant erhöhtem perioperativem Risiko, bereits präoperativer Immobilität bzw. Bettlägerigkeit („low demand“) profitieren von der alleinigen Frakturstabilisation [29]. Die Schaftrevision bei gelockerter Prothese mit oder ohne Osteosynthese sollte dennoch als Therapie der Wahl betrachtet werden [30].

Haider et al. [31] analysierten in ihrem systematischen Literaturreview 33 Studien (24 Fallserien, 9 retrospektive Arbeiten; 2509 Patienten mit B2- und B3-Frakturen). Die Patienten waren im Durchschnitt 73,8 Jahre alt (60 % Frauen). Insgesamt wurden 85,2 % der Patienten mittels Revision des femoralen Schaftes behandelt (zementiert, zementfrei, Monoblock, modular). 14,8 % erhielten alleinig eine osteosynthetische Versorgung der PPF. Es zeigte sich kein signifikanter Unterschied in Bezug auf die klinischen und radiologischen Ergebnisse zwischen beiden Kollektiven. Allerdings wurde eine höhere Revisionsrate von B3-Frakturen sowie eine höhere Sinterungsrate ohne höhere Lockerungsraten bei B2-Frakturen nach alleiniger Osteosynthese beobachtet. Die häufigsten Komplikationen kumulativ in beiden Kohorten waren die Infektion (23,6 %), die Lockerung (21,5 %) und die Re-Fraktur (16,2 %). Es zeigte sich kein Unterschied bei Erreichen der Vollbelastung. Die Mortalität war insgesamt hoch und wurde nach durchschnittlich 2,8 Jahren mit 17–21 % angegeben. Dies deckt sich mit anderen Arbeiten in denen ebenfalls vergleichsweise hohe Mortalitätsraten nach PPF aufgezeigt wurden (1 Jahr postoperativ nach zementfreier Schaftrevision 12 % vs. Osteosynthese 33 % [32]; 30-Tage-Mortalität 5,2 %, 1‑Jahres-Mortalität 21 % [33]).

In einer aktuellen Analyse des niederländischen Prothesenregisters von van Dooren et al. [34] unter Einschluss von 1879 Patienten mit erstmaliger Revision bei PPF wurde die Art der Schaftverankerung bei der Revision beleuchtet. Sowohl im zementierten als auch im zementfreien Schaftkollektiv waren die Patienten überwiegend älter als 75 Jahre (48 % vs. 47 % der Patienten). Nur 9,4 % der Patienten insgesamt waren jünger als 60 Jahre zum Zeitpunkt der PPF. Das weibliche Geschlecht überwog (70 %). In der Arbeit wurden nur Fälle eingeschlossen, welche nach erfolgter Revisionsoperation eine einheitliche Verankerung von Schaft und Pfanne hatten (zementfrei oder vollzementiert). Hybride oder auch Revers-Hybride-Versorgungen wurden ausgeschlossen. Einschränkend kommt hinzu, dass die Kollektivbeschreibung in Bezug auf die Frakturklassifikation lückenhaft war. 1621 der Patienten erhielten eine isolierte Revision des Prothesenschaftes. Die Re-Revisionsrate nach alleinigem femoralem Komponentenwechsel betrug 10 %. Bezogen auf die beiden Kollektive (555 zementiert vs. 1324 unzementiert) zeigte sich im 10-Jahres-Verlauf kein Unterschied hinsichtlich des erneuten Revisionsrisikos (zementiert 18 % vs. zementfrei 13 %) [34]. Die häufigsten Gründe für eine erneute Revision waren die Infektion (3,4 %) und die Luxation (3,3 %). Das Risiko für eine Schaftlockerung betrug bei zementierter Fixierung 3,4 % und bei zementfreier Verankerung 2,0 % (*p* = 0,1). Allerdings zeigte sich im Kollektiv ein signifikant erhöhtes Risiko der erneuten periprothetischen Fraktur (Femurschaft und/oder Azetabulum) nach zuvor zementierter Revision (3,6 % vs. 1,6 %). Das Re-Revisionsrisiko nach alleinigem Schaftwechsel bedingt durch eine erneute PPF wurde mit 1,8 % (29/1621 Patienten) beziffert.

Die Analyse des schwedischen Hüftprothesenregisters von Chatziagorou et al. [15] mit 1381 Vancouver-Typ-B-Frakturen (1064 Typ B2/3 Frakturen) konnte, wie auch die Arbeit um van Dooren et al. [34], ebenfalls keine Unterschiede zwischen zementierter und zementfreier Schaftfixierung aufzeigen. Zudem wurden von Chatziagorou et al. keine Unterschiede zwischen Monoblock (zementfrei/zementiert) und zementfreien modularen Komponenten detektiert [15]. Die Re-Revisionsraten der jeweiligen Komponenten bei den final 801 eingeschlossenen PPF Typ Vancouver B2 und B3 unterschieden sich nicht signifikant (zementfrei modular 14,4 %, zementfrei Monoblock 12,6 %, zementiert Monoblock 12,5 %). Die Patienten, welche bei der Revision eine zementierte Schaftfixierung erhielten, waren im Durschnitt älter, häufiger weiblich und wurden als Vancouver-Typ B3 klassifiziert. Das Alter zum Zeitpunkt der PPF in der „zementierten“ Kohorte lag durchschnittlich bei 80 Jahren. Die Patienten der zementfreien Kohorte (Monoblock/modular) waren zum Zeitpunkt der Frakturversorgung jünger (Ø 77,2 Jahre). Nach zementierter Schaftrevision wurde in über 90 % die schmerzadaptierte Vollbelastung freigegeben. Unabhängig von der Schaftfixierung zeigte sich nach durchschnittlich 3,6–4,1 Jahren in den jeweiligen Kollektiven eine hohe Mortalität („zementfrei modular“ 39,8 %; „zementfrei Monoblock“ 56,3 %; „zementiert“ 64,5 %) [15]. Eine orientierende Ergebnisübersicht der diskutierten Arbeiten gibt Tab. 1.

**Tab. 1:** Orientierende Ergebnisübersicht der diskutierten Studien bei der operativen Versorgung von PPF Typ Vancouver B2 und B3 (Schaftrevision vs. Osteosynthese)

	Khan et al. (2017) [27]	Di Martino et al. (2024) [28]	Haider et al. (2021) [31]	Van Dooren et al. (2023) [34]	Chatziagorou et al. (2019) [15]
Studienart	SLR	SLR	SLR	Register (Niederlande)	Register (Schweden)
Eingeschlossene Studien (*n*)	22	15	33	–	–
Studienzeitraum (Jahre)	1984–2012	1984–2022	2000–2020	2007–2021	2001–2011
Patienten (*n*)	510	1629	2509	1879	1064
Schaftrevision m/o Osteosynthese (%)	86,8	65,4	85,2	86,3	87,2
Schaftfixierung zementfrei vs. zementiert (*n*)	243 vs. 131	k. A.	k. A.	1324 vs. 555	528 vs. 273
Re-Revisionsrate nach Schaftwechsel (%)	13,1	18,5	13,5	10,0	13,5
Re-Revisionsrate nach alleiniger Osteosynthese (%)	15,4	12,7	22,9	k. A.	22,0
Mortalität (%)*	–	SR 4,1/ORIF 5,9	SR 17,2/ORIF 21,3	8,2	52,3

*SLR* Systematisches Literatur Review, *k.* *A.* eine Angabe, *ORIF* Offene Reposition und interne Fixierung, *SR* Schaftrevision

*Zu berücksichtigen ist hier der variierende Nachuntersuchungszeitraum der jeweiligen Studie

**Abb. 4:**
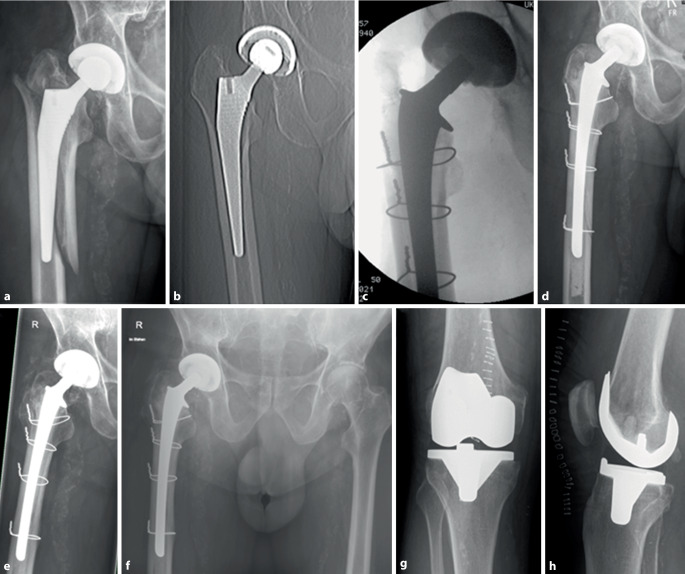
**a** 81 Jahre, männlich. Periprothetische Femurfraktur (PPF) Vancouver Typ B2 nach Sturzereignis. **b** CT-Topogramm einer früheren Untersuchung des Beckens, welches dem prätraumatischen Vergleich dient. **c** Intraoperatives Bildwandlerbild nach Osteosynthese (1,5 mm Drahtcerclage) und Schaftwechsel. **d** Postoperative Kontrolle nach Revisionsoperation mit anatomischer Frakturreposition sowie zementiertem Schaftwechsel. Postoperativ erfolgte unmittelbar die schmerzadaptierte Vollbelastung. **e/f** Röntgenaufnahme Hüftgelenk axial und Beckenübersicht. In der 1‑Jahres-Kontrolle zeigt sich eine konsolidierte Fraktur. **g/h** Röntgen Kniegelenk a.p. und seitlich. Bei guter postoperativer Mobilität und ipsilateral fortgeschrittener Gonarthrose erfolgte 1,5 Jahre nach der PPF die Implantation eines bikondylären Oberflächenersatzes

Im Allgemeinen sind qualitativ hochwertige Arbeiten mit hohem Evidenzgrad hinsichtlich der zu favorisierenden Schaftfixierung bei PPF rar. Viele der Arbeiten sind Fallserien oder retrospektive Studien mit zum Teil geringer Fallzahl. Sponer et al. untersuchten beispielsweise in ihrer Arbeit 37 Patienten (21 zementiert vs. 16 unzementiert) [35]. Die Patienten mit zementierter Schaftrevision wurden postoperativ schmerzadaptiert voll belastet. Die Frakturkonsolidierung innerhalb des Kollektivs wurde mit 91,9 % beziffert [35]. Axenhus et al. [36] analysierten in einer retrospektiven Fallserie (Evidenzlevel III) von 241 Patienten das Ergebnis nach zementierter (*n* = 112) und unzementierter (*n* = 129) Schaftrevision nach Vancouver-B2- und -B3-Fraktur. Die Patienten mit einem zementierten Schaftwechsel waren älter und vorwiegend weiblich. Der Nachuntersuchungszeitraum betrug im Mittel 3,9 Jahre für die zementierte Gruppe und 5,5 Jahre für die unzementierten Schaftrevisionen. In der zementfreien Gruppe zeigte sich eine signifikant erhöhte Lockerungsrate (9,3 % vs. 0,6 %) sowie eine im Vergleich längere Zeit bis zur Frakturkonsolidierung (16,7 Wochen vs. 11,4 Wochen). Die Mortalität im zementierten Kollektiv (Ø 83 Jahre) war signifikant höher (30-Tage-Mortalität 7,1 % vs. 1,6 %; 1 Jahr 15,2 % vs. 11,6 %) als im durchschnittlich einige Jahre jüngeren zementfreien Kollektiv (Ø 76 Jahre). Auch andere aktuelle Arbeiten konnten aufzeigen, dass die zementierte Schaftrevision nach anatomischer Frakturreposition von Vancouver-Typ-B2- und -B3-Frakturen die zügige Mobilisation des Patienten ermöglicht, ohne die Frakturkonsolidierung zu kompromittieren [37]. Der zementierte Schaftwechsel ist bei betagten Patienten in Zusammenschau der Literatur durchaus ein probates Verfahren, welches mit zufriedenstellenden Ergebnissen einhergeht ([30]; Abb. 4a–h).

## Operatives Vorgehen – Ist die Zement-in-Zement-Revision eine Option?

Als alternative Versorgungsstrategie wird in einigen Arbeiten auch eine Zement-in-Zement-Revision beschrieben [38–40]. Der vermeintliche Vorteil liegt in einer kürzeren Operationszeit, weniger Komplikationen, weniger Sinterung und keinem Unterschied in Bezug auf die Re-Revisionsrate [38, 40]. Limitierend bei der Interpretation der Daten sind allerdings die wenigen verfügbaren Studien und die eher geringen Fallzahlen. Die weitestgehende Unversehrtheit des Zementmantels, welche als Voraussetzung für eine Zement-in-Zement-Revision angesehen werden kann, damit letztendlich auch die Frakturreposition gelingt, ist essenziell [39].

**Abb. 5:**
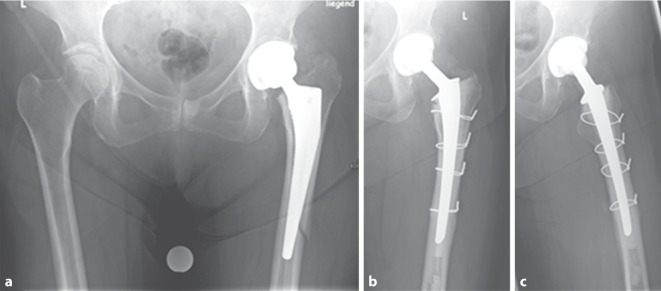
**a** 70 Jahre, weiblich. Eine Woche nach Implantation einer zementfreien Hüfttotalendoprothese immobilisierende Schmerzen bei der Mobilisation im Rahmen der Rehabilitation. Konventionell radiologischer Nachweis einer periprothetischen Femurfraktur mit Sinterung des Schaftes (Vancouver Typ B2). Tendenziell breiterer Markraum. **b/c** Röntgen Hüftegelenk a.p. und axial. Nach Revisionsoperation mit offener Reposition und Osteosynthese (1,5 mm Drahtcerclagen) sowie zementiertem Schaftwechsel zeigt sich in der Nachuntersuchung eine konsolidierte Fraktur. Postoperativ erfolgte unmittelbar die schmerzadaptierte Vollbelastung

Grundsätzlich sollte im Fall einer PPF ein individualisierter Therapieansatz gewählt werden. Verschiedene Umfeldfaktoren, Komorbiditäten und die bekannten Risikofaktoren für eine PPF sind zu berücksichtigen. Kommt es bereits im frühen postoperativen Verlauf zu einer PPF – gegebenenfalls auch ohne Anhalt für ein adäquates Trauma – sollte im Falle der primär zementfreien Schaftfixierung bei existierenden Risikofaktoren (z. B. Alter, weibliches Geschlecht, Femurkonfiguration Typ Dorr C, Osteopenie/Osteoporose) das Verankerungsprinzip bei der Revision überdacht werden (Abb. 5**a-c**).

**Abb. 6:**
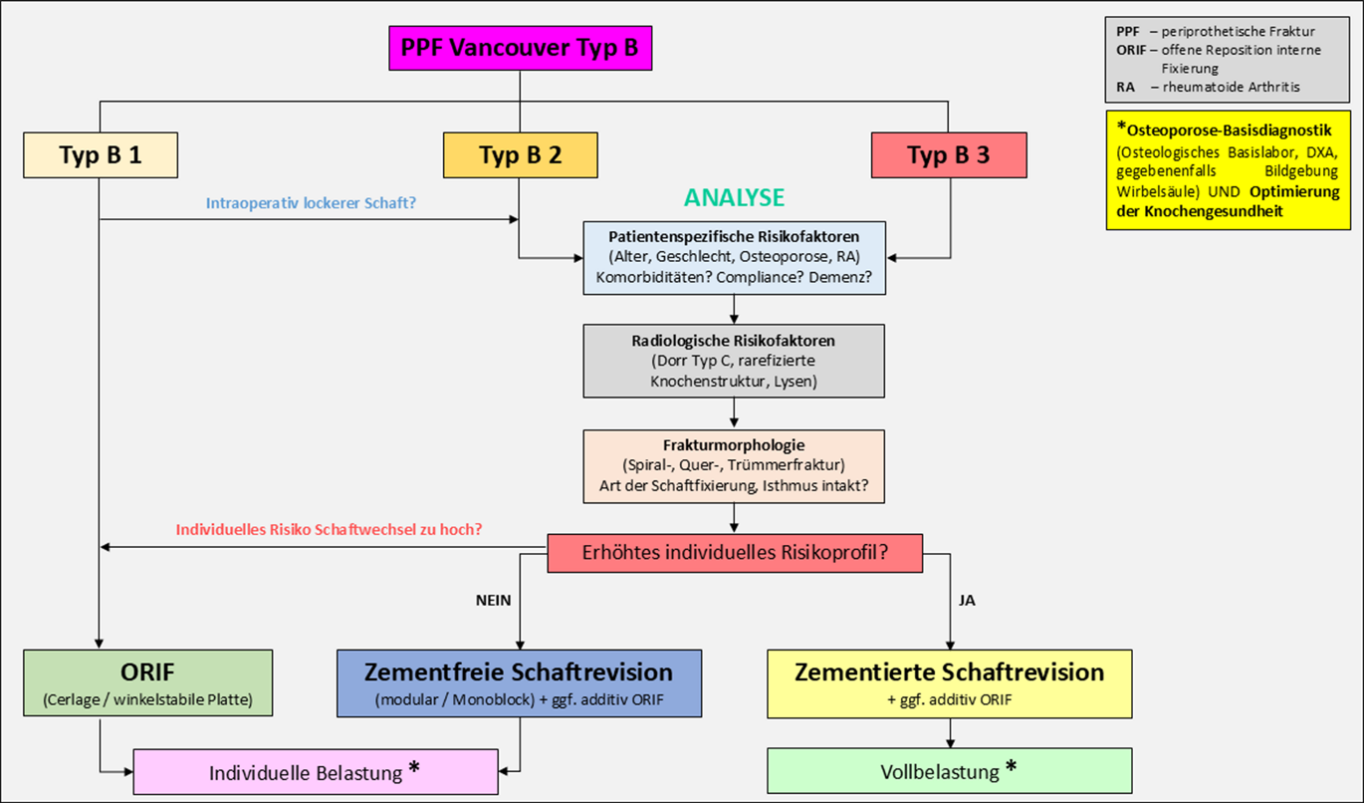
Orientierende Handlungsübersicht bei periprothetischer Femurfraktur (*PPF*) Vancouver Typ B

Als orientierende Handlungsübersicht bei PPF Vancouver Typ B dient Abb. 6.

## Fazit für die Praxis

- Die Mortalität nach PPF ist unabhängig von der gewählten Therapie hoch.
- Alter und Knochenqualität spielen eine Rolle bei der operativen Versorgung.
- Zementierte und zementfreie Schäfte werden in der Literatur häufig verwendet. Signifikante Unterschiede zeigen sich nicht.
- Der Vorteil von zementfreien Schäften in modularer Ausfertigung wird von der Literatur aktuell nicht gestützt.
- Zement-in-Zement-Revisionen zeigen bei hochbetagten Patienten gute Ergebnisse, wenngleich wenige Arbeiten mit nur kleinen Fallzahlen existieren.
- Unter Berücksichtigung der Umfeldfaktoren und Komorbiditäten empfiehlt sich ein individueller Ansatz bei der Versorgung PPF.
- Nach Möglichkeit sollten Spezialisten aus Traumatologie und Orthopädie gemeinsam agieren, um die bestmögliche Versorgung sicherzustellen.
- Beim geriatrischen Patienten sollte zur Vermeidung von Komplikationen postoperativ eine Vollbelastung angestrebt werden.

## Abkürzungen

a.p.: anterior-posterior
DXA: Dual Energy X-ray Absorptiometry
EPRD: Endoprothesenregister Deutschland
k. A.: keine Angabe
ORIF: Offene Reposition und interne Fixierung
PPF: periprothetische Femurfraktur
RA: Rheumatoide Arthritis
SLR: Systematisches Literatur Review
SR: Schaftrevision
UCS: Unified Classification System
V.a.: Verdacht auf
z.B.: zum Beispiel
